# Interferon Beta-1a Improves Urinary Symptoms, Reduces Proviral Load, and Modifies the Immune Response in a Patient with HAM/TSP

**DOI:** 10.1155/2012/958786

**Published:** 2012-08-16

**Authors:** Davi Tanajura Costa, Michael Sundberg, Lúcia Passos, André Luiz Muniz, Silvane Santos

**Affiliations:** Serviço de Imunologia, Hospital Universitário Prof. Edgard Santos, 5 Andar, Rua João das Botas S/N, Canela, 40110-160 Salvador, BA, Brazil

## Abstract

The human T-cell lymphotropic virus type 1 (HTLV-1) is the known causative agent of a chronic neurologic condition known as HTLV-1-associated myelopathy/tropical spastic paraparesis (HAM/TSP). Although several therapies have been evaluated for HAM/TSP, none have been approved for use in humans. In this paper, we describe a 55-year-old female patient with HAM/TSP who was treated with interferon beta-1a. This patient, in comparison to 20 female patients with HAM/TSP who were not treated, showed improvement in urinary symptoms over four years of therapy, as well as a reduction in HTLV-1 proviral load and serum cytokine levels typically observed in HAM/TSP. This improved outcome merits further controlled studies on the use and efficacy of interferon beta-1a as a therapy for HAM/TSP.

## 1. Introduction

HTLV-1-associated myelopathy/tropical spastic paraparesis (HAM/TSP) is a chronic, insidious neurologic disease that presents with impairment of lower limb strength, autonomic abnormalities including neurogenic bladder and bowel, and mild changes in sensation [1]. Currently, there are no specific treatments for this condition, and therapy is guided by supportive measures for associated urinary symptoms, pain, and spasticity [2]. In this paper, we present a HAM/TSP case treated with interferon beta-1a with a good outcome, compared to age- and gender-matched patients also with HAM/TSP who were not treated.

## 2. Case Report

The patient is a 55-year-old woman who first presented to our HTLV clinic in 2001 with insidious paraparesis and urinary symptoms including urgency, incontinence, and nocturia, since 1999. She was referred to our service by a local neurologist after noting a positive HTLV-1 serology. The patient's medical history includes hypothyroidism and positive serology for hepatitis C. At the time of presentation, the patient was taking vitamin C 2.0 g daily, 1-thyroxin 75 mcg daily, and oxybutynin 5 mg twice daily.

On routine neurologic examination at presentation, the patient was noted to have grade III proximal and distal strength (Medical Research Council [3]), with increased tone. Reflexes were grade III (Campbell [4]) at the biceps, triceps, and brachioradialis, and grade IV at the patella and ankle. Bilateral Babinski sign was observed. The patient's laboratory analyses were unremarkable except for elevated liver enzymes, including AST (76 g/L, normal 5–40 g/L) and ALT (99 g/L, normal 7–56 g/L). Measurements of IFN-gamma and IL-10 at the time were 10750 pg/mL and 60 pg/mL, respectively. In the first year of presentation, the patient was started on 30 mcg (6 million units) of interferon beta-1a (Avonex, Biogen Idec) every 15 days by intramuscular injection. The final dose of 30 mcg every week was not reached because of the patient's complaints of joint pain and headache. 

**Figure 1:**
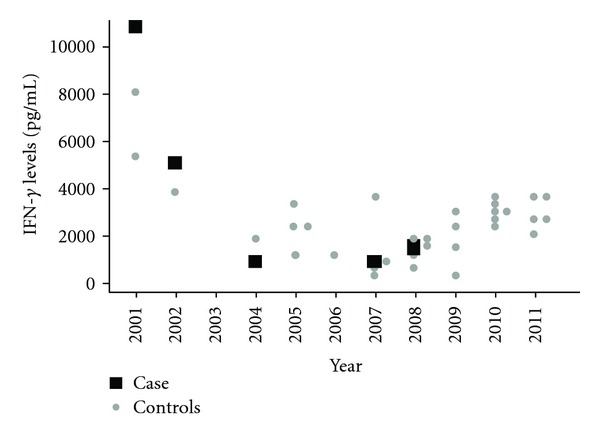
IFN-gamma levels changes over time.

**Figure 2:**
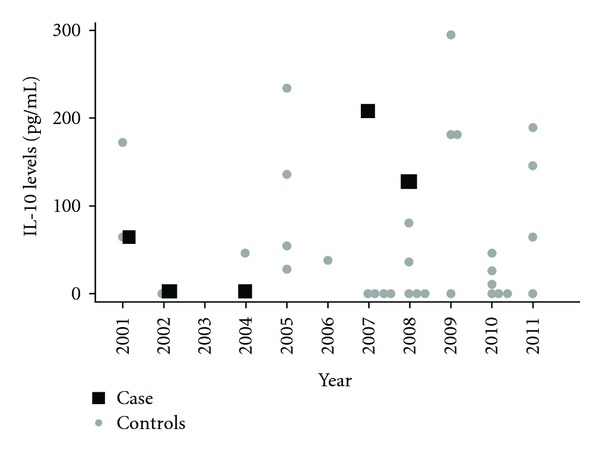
IL-10 levels changes over time.

The patient was reevaluated regularly over the course of the next seven years. Within the first three years of treatment, the patient reported improved urinary symptoms, including no further daytime urgency nor incontinence, and decreased nocturia. However, she did not notice a change in her motor symptoms. In 2007, six years after initial presentation, the patient's first proviral load was measured at 180 copies/10^6^ cells (log 2.26). She continued using interferon beta-1a, and in 2008 the proviral load was undetectable. Liver enzymes, which had been measured annually, also returned to normal at that time. IFN-gamma and IL-10 levels, which were evaluated before and after the start of interferon beta-1a, are described in Figures 1 and 2, respectively. Initially, IFN-gamma decreased to 5170 pg/mL in the first year of treatment. It then further declined to 906 pg/mL by the sixth year of treatment, followed by a small increase to 1783 pg/mL in the seventh year of treatment. IL-10 levels were undetectable in the years after commencing treatment, and rose slightly to 204 and 126 pg/mL on the last two measurements. During this time, the patient's urinary symptoms improved and motor symptoms did not progress.

To compare cytokine and proviral load levels, we selected 20 controls from an ongoing cohort study that were matched by gender and age to our case. All patients had HAM/TSP and had never received corticosteroids, immune suppressors, or immune modulator treatments. Cytokine levels for controls are also represented in Figures 1 and 2 with corresponding time measurement. Cytokine levels for all 20 individuals were measured at least once (initial determination); second measurements were taken later in time from 10 individuals for IFN-gamma and eight individuals for IL-10 (initial and final determination). For the proviral load, 17 individuals had at least one proviral load determination (initial determination) and in this group, 4 had at least two proviral loads determinations at different times (initial and final determination). 

Initial mean for IFN gamma was 2261.7 ± 1890.3 pg/mL (*n* = 20) and final mean was 2735.5 ± 897.15 pg/mL (*n* = 10) and were equal when compared (*P* = 0.1, sign test). For IL-10, initial mean was 68.2 ± 85.4 pg/mL (*n* = 20) and final 76.9 ± 89.1 pg/mL (*n* = 8), and the groups were equal when compared (*P* = 1, sign test). Initial control group proviral load mean was 205,999.2 ± 115,201.2 copies/10^6^ peripheral blood mononuclear cells (PBMCs) (*n* = 17) and final mean 111,333.7 ± 76,669.8 copies/10^6^ PBMCs (*n* = 4), and the groups were equal when compared (*P* = 1, sign test).

Patients described in this study were part of a cohort study and all provided written consent. Approval was granted by a local committee in ethics and research. 

## 3. Discussion

Several different therapies have been considered and evaluated for HAM/TSP with some promise, although none have yet been approved for use in patients. One randomized, placebo-controlled trial evaluated the use of zidovudine with lamivudine in HAM/TSP, but failed to show a difference in clinical or immunologic markers between groups [5]. In an open trial evaluating different doses of interferon alpha in HAM/TSP patients, significant changes were detected in both motor and urinary scores with high doses of the medication [6]. Other noncontrolled studies using interferon alpha in patients with HAM/TSP have observed better clinical outcomes and a reduction in proviral load [7–9], but no placebo randomized controlled trial has been conducted with this treatment and long-term studies have failed to show clear benefit [8].

Interferon beta-1a has been used to treat multiple sclerosis in the relapsing-remitting form and has proven to be beneficial in several trials [10, 11]. In HAM/TSP, only one study has evaluated the treatment using a dose of 60 mcg twice per week. In this study, a reduction of tax-CD8+ cells was noted, but not a reduction in the proviral DNA load [12]. In our case, we chose to treat with interferon beta-1a instead of interferon alpha because of a lack of benefits exhibited in long-term studies, because of the availability of interferon beta-1a, and because interferon alpha has a daily dosage schedule instead of the simple, weekly dosage of interferon beta-1a. Our patient utilized a lower dose at 30 mcg every 15 days. However, we noted good outcomes and a good tolerance with administration at this amount and frequency. 

To compare to others not receiving immune modulation treatment, we looked at proviral load, the inflammatory cytokine IFN-gamma, and the regulatory cytokine IL-10 in comparison to 20 control patients from an ongoing cohort study. Levels of proviral load and IFN-gamma stayed in the low range in our case, especially after interferon beta-1a use, showing a reduction in inflammation mechanisms. Also, IL-10, an anti-inflammatory cytokine, was very low in the beginning but increased after the use of interferon beta-1a. For controls, there was no statistical difference between the first and last dosage for cytokines and proviral load, despite the low sample. Previous research has pointed out that proviral load does not change statistically over time, despite some variation in levels [13, 14]. However, others point to the variation in the proviral load over time [15]. Furthermore, in another work, cytokines also remain relatively constant [16]. 

In our patient, improved urinary symptoms were noted, but motor symptoms were unchanged. One possible explanation is that spinal cord atrophy—which is frequently seen in patients with HAM/TSP—is more predominant in the anterior horn motor tracts important for lower-limb movement than in the lateral and intermediate columns where autonomic bladder nerve tracts run. 

We conclude that interferon beta-1a is a therapeutic option for HAM/TSP and controlled trials are needed to evaluate this drug.
